# Induction of AmpC-Mediated β-Lactam Resistance Requires a Single Lytic Transglycosylase in Agrobacterium tumefaciens

**DOI:** 10.1128/aem.00333-22

**Published:** 2022-05-31

**Authors:** Wanda M. Figueroa-Cuilan, Matthew Howell, Christopher Richards, Amelia Randich, Akhilesh K. Yadav, Felipe Cava, Pamela J. B. Brown

**Affiliations:** a Division of Biological Sciences, University of Missouri –Columbia, Columbia, Missouri, USA; b Laboratory for Molecular Infection Medicine, Department of Molecular Biology, Umeå University, Umeå, Sweden; Norwegian University of Life Sciences

**Keywords:** antibiotic resistance, *Agrobacterium tumefaciens*, β-lactamases, anhydro amidases, membrane-bound lytic transglycosylases, ampicillin, plant transformation

## Abstract

The remarkable ability of Agrobacterium tumefaciens to transfer DNA to plant cells has allowed the generation of important transgenic crops. One challenge of A. tumefaciens-mediated transformation is eliminating the bacteria after plant transformation to prevent detrimental effects to plants and the release of engineered bacteria to the environment. Here, we use a reverse-genetics approach to identify genes involved in ampicillin resistance, with the goal of utilizing these antibiotic-sensitive strains for plant transformations. We show that treating A. tumefaciens C58 with ampicillin led to increased β-lactamase production, a response dependent on the broad-spectrum β-lactamase AmpC and its transcription factor, AmpR. Loss of the putative *ampD* orthologue *atu2113* led to constitutive production of AmpC-dependent β-lactamase activity and ampicillin resistance. Finally, one cell wall remodeling enzyme, MltB3, was necessary for the AmpC-dependent β-lactamase activity, and its loss elicited ampicillin and carbenicillin sensitivity in the A. tumefaciens C58 and GV3101 strains. Furthermore, GV3101 Δ*mltB3* transforms plants with efficiency comparable to that of the wild type but can be cleared with sublethal concentrations of ampicillin. The functional characterization of the genes involved in the inducible ampicillin resistance pathway of A. tumefaciens constitutes a major step forward in efforts to reduce the intrinsic antibiotic resistance of this bacterium.

**IMPORTANCE** Agrobacterium tumefaciens, a significant biotechnological tool for production of transgenic plant lines, is highly resistant to a wide variety of antibiotics, posing challenges for various applications. One challenge is the efficient elimination of A. tumefaciens from transformed plant tissue without using levels of antibiotics that are toxic to the plants. Here, we present the functional characterization of genes involved in β-lactam resistance in A. tumefaciens. Knowledge about proteins that promote or inhibit β-lactam resistance will enable the development of strains to improve the efficiency of *Agrobacterium-*mediated plant genetic transformations. Effective removal of *Agrobacterium* from transformed plant tissue has the potential to maximize crop yield and food production, improving the outlook for global food security.

## INTRODUCTION

*Rhizobiaceae* is a family of bacteria that includes soil-dwelling and plant-associated bacteria. While some species of this family have the ability to establish symbiotic relationships with plants, others are pathogenic, such as the genus *Agrobacterium*. Members of this genus are responsible for a number of diseases, including cane gall disease (Agrobacterium rubi), hairy root disease (Agrobacterium rhizogenes), crown gall disease of grapes (Agrobacterium vitis), and crown gall disease to flowering plants and woody shrubs (Agrobacterium tumefaciens) (1–5). In nature, A. tumefaciens causes crown gall by adhering to wounded plants and injecting a section of a bacterial DNA plasmid (transfer DNA [tDNA]) that integrates into the plant chromosomes (1–3, 6–11). Expression of genes on the tDNA segment causes the plant to produce custom energy sources that only *Agrobacterium* can use (9, 10). The phytohormones encoded on the tDNA lead to overproliferation of the host plant cells and eventual gall formation (6). Gall formation on plants and trees leads to crop damage, and significant economic losses have been attributed to this issue every year (2, 3).

While the genus *Agrobacterium* exhibits pathogenicity against plants, the natural ability of *Agrobacterium* to transfer DNA to plants has been exploited to produce transgenic plants through genetic engineering (5, 6, 9, 11, 12). However, one challenge for A. tumefaciens-mediated plant transformations is the elimination of the bacteria from the transformed plant tissue. Elimination of recombinant A. tumefaciens from plant tissues is crucial to prevent detrimental effects for plants and to reduce the risk of releasing engineered bacteria into the environment (13–15). β-Lactam antibiotics are frequently applied during plant transformations to eliminate A. tumefaciens from plant tissues and are preferred over other classes of antibiotics (16–18). Because β-lactams target cell wall synthesis, a process unique to bacteria, they are less toxic to eukaryotic plant cells than antibiotics that inhibit protein or nucleic acid synthesis (19, 20). However, the natural resistance of A. tumefaciens to β-lactams can be overcome only with toxic levels (~200 to 1,000 mg/L), which has been shown to cause embryogenic tissue necrosis or to affect plant tissue growth and regeneration rates in a wide variety of plants (16, 17, 21–26). Moreover, depending on the concentration and class of β-lactam, clearing *Agrobacterium* from embryos can take up to 60 days, yet, in some cases, complete elimination of A. tumefaciens is not achieved (27). Thus, currently, there is a need for the identification and understanding of regulatory pathways and enzymes involved in β-lactam resistance in A. tumefaciens. Functional characterization of bacterial enzymes involved in β-lactam resistance will permit the development of tools that could improve the efficiency of plant genetic transformations and therefore maximize crop yields and food production.

β-Lactam antibiotics target the bacterial cell wall by inhibiting the activity of penicillin binding proteins (PBPs), the enzymes involved in the synthesis of the bacterial peptidoglycan (PG) cell wall (28–35). The bacterial PG cell wall is an essential polymer consisting of alternating *N-*acetylglucosamine (GlcNAc) and *N-*acetylmuramic acid (MurNAc) sugars cross-linked through peptide bridges (36–41). Because the PG cell wall is a covalently enclosed polymer, its expansion requires not only cell wall synthesis but also remodeling. Cell wall remodeling is mediated by PG degradation enzymes such as the lytic transglycosylases (LTs) (42–45). To allow cell wall expansion, LTs cleave between the MurNAc and GlcNAc sugar strands, resulting in the formation of 1,6-anhydroMurNAc GlcNAc on glycan strands and the liberation of 1,6-anhydromuropeptide (Anh-Mur) cell wall degradation fragments. The liberated Anh-Mur fragments are transported to the bacterial cytoplasm for cell wall recycling (39, 46, 47, 48). In the cytoplasm, the recycling of Anh-Mur fragments keeps the concentration of these products low (46, 48–51). However, cell wall stressors such as treatment with β-lactam antibiotics or mutations that inhibit the cell wall recycling pathway result in the accumulation of Anh-Mur cell wall degradation fragments and derepression of β-lactamases (48, 51–55). In bacteria including Pseudomonas aeruginosa and Enterobacter cloacae, the Anh-Mur cell wall degradation fragments are transcriptional activators of inducible β-lactamases, which are enzymes that cleave and inactivate β-lactam antibiotics (47, 53, 56–61).

**FIG 1:**
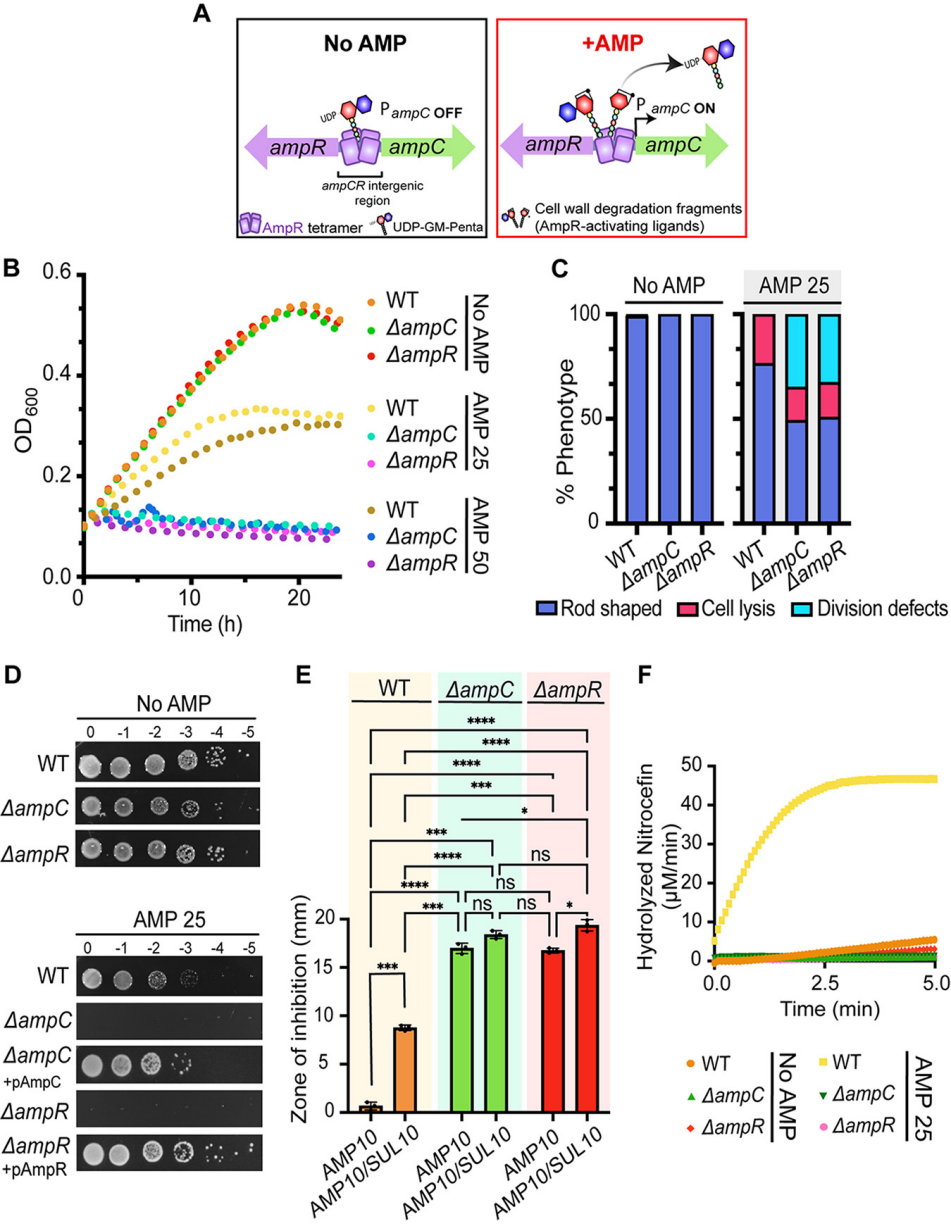
The AmpC-AmpR operon is responsible for induced ampicillin resistance in Agrobacterium tumefaciens C58. (A) Operon organization and proposed ampicillin resistance mechanism. (Left, No AMP) Briefly, in the absence of β-lactams such as ampicillin (AMP), *ampC* expression is repressed by AmpR. AmpR-mediated repression is maintained as long as the AmpR-inactivating ligand, UDP-GM-pentapeptide, is bound to AmpR (P*ampC* OFF). (Right, +AMP) In contrast, the presence of ampicillin (AMP) increases the pools of AmpR-activating ligands or the cell wall degradation fragments (anhydro modification is depicted by a ring), which are known to displace AmpR-inactivating ligands. As a result, the increase in AmpR-activating ligands activates AmpR and *ampC* is transcribed (P*ampC* ON). (B) Growth of A. tumefaciens WT, Δ*ampC*, and Δ*ampR* cells in the absence (No AMP) and presence of ampicillin at 25 or 50 μg/mL (AMP 25 or AMP 50, respectively) for 24 h (*n* = 1; 2 replicates). (C) Quantitative analysis of phase-contrast microscopy of exponentially growing strains in the absence (No AMP) or presence of ampicillin at 25 μg/mL (AMP 25). The percent phenotype was calculated by counting the number of cells displaying one of the phenotypes indicated (1 cell = 1 phenotype) and dividing it by the total number of cells per strain. (D) Ampicillin susceptibility assay performed by spotting dilutions. Briefly, exponential cultures were serially diluted, spotted on LB solid medium containing no ampicillin (No AMP) or ampicillin at 25 μg/mL (AMP 25), and incubated at 28°C for 36 h before imaging. Plates used to demonstrate complementation (Δ*ampC* + pAmpC or Δ*ampR* + pAmpR) included 1 μM IPTG to induce expression of plasmid-encoded AmpC or AmpR. (E) Disk susceptibility was performed on lawns of the indicated strains grown on LB plates for 24 h at 28°C (*n* = 2). AMP 10, disk containing 10 μg/mL of ampicillin; AMP 10/SUL 10, disk containing 10 μg/mL of ampicillin and 10 μg/mL of sulbactam, a broad-spectrum β-lactamase inhibitor. Data represent the mean (±standard deviation [SD]) of three independent experiments. ****, *P < *0.0001; ***, *P < *0.001; **, *P < *0.01; *, *P <* 0.1; ns, not significant. (F) Determination of β-lactamase production performed by a nitrocefin assay using cell lysates. No AMP or AMP 25 indicates cells untreated or treated with ampicillin at 25 μg/mL, respectively, for 2 h before the generation of cell lysates. The data shown represent one of two biological replicates.

In the soil environment, many soil microorganisms produce antibiotics to compete for survival, selecting for intrinsic resistance pathways in soil pathogens. For example, the genomes of many soil bacteria contain β-lactamases, such as the cephalosporinase AmpC (53, 62). As a cephalosporinase, AmpC is known to destroy β-lactam antibiotics, including monobactams, cephalosporins, and penicillins (53). The AmpC consensus protein sequence consists of a signal sequence for periplasmic transport and a β-lactamase catalytic domain (see Fig. S1A in the supplemental material). The regulation of AmpC expression varies across bacteria. In Escherichia coli, AmpC is a noninducible β-lactamase that is expressed at low levels and regulated by a promoter and a growth rate-dependent attenuator mechanism (63–65). In contrast, in P. aeruginosa and some enterobacteria, AmpC is normally expressed at low levels but is inducible and can be derepressed during exposure to β-lactams (53, 58, 60, 61, 66). In these cases, AmpC expression is regulated by AmpR, a LysR-type transcriptional regulator found encoded in an operon with AmpC (57, 58, 60, 61, 62, 66). AmpR consists of two domains: a helix-turn-helix DNA-binding domain (DBD), which binds the intergenic region between AmpC and AmpR, and a LysR effector-binding domain (EBD), which contains the regulatory region of AmpR (Fig. 1A; Fig. S1A) (58, 59). AmpR is a bifunctional transcriptional regulator that controls both the activation and repression of AmpC. The induction mechanism of AmpC by AmpR in response to β-lactams is linked to bacterial cell wall synthesis, remodeling, and recycling (48, 51, 53, 56, 60, 67). Indeed, the Anh-Mur cell wall degradation fragments released by LTs during cell growth are AmpR-activating molecules. In contrast, cell wall building blocks such as UDP-GlcNAc MurNAc pentapeptides bind to AmpR and repress *ampC* transcription (Fig. 1A). A block in bacterial cell wall synthesis after exposure to β-lactams results in accumulation of Anh-Mur cell wall degradation products in the bacterial cytoplasm, displacement of the AmpR repressor UDP-GlcNAc MurNAc pentapeptide, activation of AmpR, and transcription of *ampC* (Fig. 1A).

The genus *Agrobacterium* is naturally resistant to β-lactams, and the molecular basis for this resistance is poorly understood. A study that screened for β-lactamase production in A. tumefaciens detected cephalosporinase production (68) and identified one putative cephalosporinase gene, an AmpC homolog (*atu3077*), in the A. tumefaciens genome. In addition, similar to *Enterobacteriaceae* and *Pseudomonadales*, *ampC* is found adjacent to a divergently transcribed *ampR* gene in most *Agrobacterium* genomes (Fig. 1A). Here, we sought to determine if AmpC is a functional β-lactamase, if AmpC is inducible, and if the natural ampicillin resistance observed in A. tumefaciens is dependent on AmpC.

We present a functional characterization of proteins involved in intrinsic ampicillin resistance in A. tumefaciens. We found that AmpR is required for AmpC-dependent β-lactamase activity and that loss of the anhydro-amidase AmpD (*atu2113*, misannotated as an AmiD homolog in the genome [69]) leads to increased resistance to ampicillin, a process dependent on AmpC. We suggest that AmpD is required for proper recycling of cell wall degradation products and its loss results in the accumulation of cell wall degradation products and activation of AmpC by AmpR. Furthermore, we found that a single LT, the membrane-bound lytic transglycosylase B3 (MltB3), is necessary for AmpC-dependent β-lactamase activity and that its loss leads to ampicillin sensitivity in the A. tumefaciens strains C58 and GV3101. Finally, transformation of Arabidopsis thaliana utilizing a Δ*mltB3* GV3101 strain requires significantly lower concentrations of ampicillin while exhibiting similar wild-type (WT) transformation efficiency. This work underscores the significance of understanding the β-lactam resistance pathway of A. tumefaciens with the aim of expanding tools for the A. tumefaciens*-*mediated transformations.

## RESULTS AND DISCUSSION

### The AmpC-AmpR operon is responsible for inducible ampicillin resistance in A. tumefaciens C58.

To begin our characterization, we first assessed the susceptibility of A. tumefaciens to different concentrations of ampicillin near the MIC reported for A. tumefaciens on solid and liquid media (70). We found that cells grown in LB medium with 25 or 50 μg/mL ampicillin (AMP 25 or AMP 50, respectively) for 24 h displayed slow growth in liquid medium in comparison to cells grown in LB medium without ampicillin (LB No AMP) (Fig. 1B). To better understand the cause of this growth defect, we performed phase-contrast microscopy of cells treated with AMP 25 for 2 h (see Fig. S1B in the supplemental material). We found that treatment with AMP 25 causes a significant increase in the median cell length (Fig. S1C) and that 23.5% of the cells underwent cell lysis (Fig. 1C; Fig. S1B), confirming that the bactericidal effect of AMP 25 on WT A. tumefaciens is the cause of the overall decrease in optical density. Similarly, WT cells grown on AMP 25 solid medium for 36 h have a viability defect in comparison to WT cells grown in LB No AMP (Fig. 1D). The increased sensitivity of WT A. tumefaciens to ampicillin in the presence of sulbactam, a broad-spectrum β-lactamase inhibitor, suggests that β-lactamase production is responsible for the observed ampicillin resistance (Fig. 1E). Finally, to determine if β-lactamase production is induced, we treated WT cells with AMP 25 for 2 h, generated whole-cell lysates, and performed nitrocefin assays on total protein content (Fig. 1F). Nitrocefin is a chromogenic substrate related to the cephalosporins that undergoes a color change when it is hydrolyzed by β-lactamases (71). After treatment of WT cells with AMP 25 for 2 h, the activity of β-lactamases was readily detected in lysates by using nitrocefin assays (Fig. 1F). Together, these results suggest that A. tumefaciens C58 β-lactamase production is induced in the presence of β-lactams such as ampicillin. To assess the contributions of putative enzymes involved in ampicillin resistance, we employed a reverse-genetics approach (72).

The *ampC* ortholog of A. tumefaciens C58 (*atu3077*) is present on the linear chromosome and is the only putative inducible β-lactamase gene in the genome of A. tumefaciens C58 (53). *ampC* is syntenic with *ampR*, and the A. tumefaciens AmpC and AmpR proteins are 74.7% and 85.9% similar to their respective orthologs from P. aeruginosa. To determine the role of AmpC, we deleted *ampC* (*atu3077*) from the A. tumefaciens C58 genome. Deletion of *ampC* does not have a major impact on cell growth and cell viability (Fig. 1B and D) or cell morphology (Fig. S1B), beyond a slight increase in cell length (Fig. S1C). To pinpoint the contribution of *ampC* to ampicillin resistance, we assessed the growth dynamics of Δ*ampC* cells in the presence of AMP in liquid medium (Fig. 1B). Δ*ampC* cells treated with AMP 25 or AMP 50 for 24 h show a severe growth defect, indicating that AmpC contributes to ampicillin resistance. Similarly, deletion of *ampC* results in a severe growth viability defect on solid medium containing AMP 25 (Fig. 1D). Production of plasmid-encoded AmpC in Δ*ampC* cells restores growth and viability in the presence of AMP 25 (Fig. 1D). In addition, Δ*ampC* cells treated with AMP 25 exhibit cell division defects (34.8%) and cell lysis (15.8%) (Fig. 1C; Fig. S1B).

To confirm that ampicillin resistance is mediated by the AmpC β-lactamase, we used the disk diffusion assay to compare levels of resistance to ampicillin in the presence and absence of the broad-spectrum β-lactamase inhibitor sulbactam (Fig. 1E). As expected, Δ*ampC* leads to increased sensitivity to ampicillin, and the presence of sulbactam does not result in large increases in the zone of growth inhibition (Fig. 1E). Furthermore, monitoring the rates of nitrocefin hydrolysis shows that production of β-lactamase is readily detected in WT cells treated with AMP 25 but is undetectable in Δ*ampC* cells following AMP 25 treatment (Fig. 1F). Together, these observations suggest that the natural resistance to ampicillin depends on the presence of AmpC, which functions as an inducible β-lactamase.

We hypothesized that if transcription of *ampC* is strictly controlled by AmpR, deletion of *ampR* should mimic deletion of *ampC.* To test this hypothesis, we deleted *ampR* (*atu3078*) from the genome of A. tumefaciens C58. Deletion of *ampR* does not have a major impact on cell growth (Fig. 1B), morphology (Fig. S1B), cell length (Fig. S1C), or cell viability (Fig. 1D) in LB medium. Low concentrations of ampicillin in either liquid or solid medium are lethal to Δ*ampR* cells and result in cell division defects and cell lysis similar to those of Δ*ampC* cells (Fig. 1B and C; Fig. S1B). Production of plasmid-encoded AmpR restores the viability of Δ*ampR* cells on solid medium with AMP 25 (Fig. 1D). Like Δ*ampC* cells, Δ*ampR* cells fail to produce detectable β-lactamase activity when treated with AMP 25 (Fig. 1F). Together, these results suggest that AmpR and AmpC contribute to ampicillin resistance in A. tumefaciens. Based on agreement with the general mechanism of characterized AmpR-AmpC pathways, we hypothesize that AmpR is necessary for induction of the AmpC β-lactamase in the presence of ampicillin.

### Loss of AmpD derepresses β-lactamases in A. tumefaciens C58.

The finding that AmpC and AmpR are necessary for ampicillin resistance in A. tumefaciens C58 led us to explore how the pools of different cell wall fragments alter AmpC-mediated β-lactamase induction. Similar to exposure to β-lactams, loss of cell wall recycling amidases has been shown to increase the AmpR-activating fragments (cell wall degradation fragments) in the cytoplasm, resulting in the transcriptional derepression of *ampC* and β-lactam resistance (45, 54, 73–76). The genome of A. tumefaciens contains one 1,6-anhydro amidase ortholog, *atu2113*, reannotated here as *ampD*. The domain organization of AmpD consists of the Amidase_2 (Ami_2) catalytic domain and a PG-binding domain (PBD) that facilitates the interaction with cell wall products (Fig. S2A) (44, 47). A. tumefaciens AmpD exhibits 64.4% sequence similarity to AmpDh2, one of three broad-spectrum 1,6-anhydro amidase AmpDh paralogs found in Pseudomonas aeruginosa (53–55, 77).

**FIG 2:**
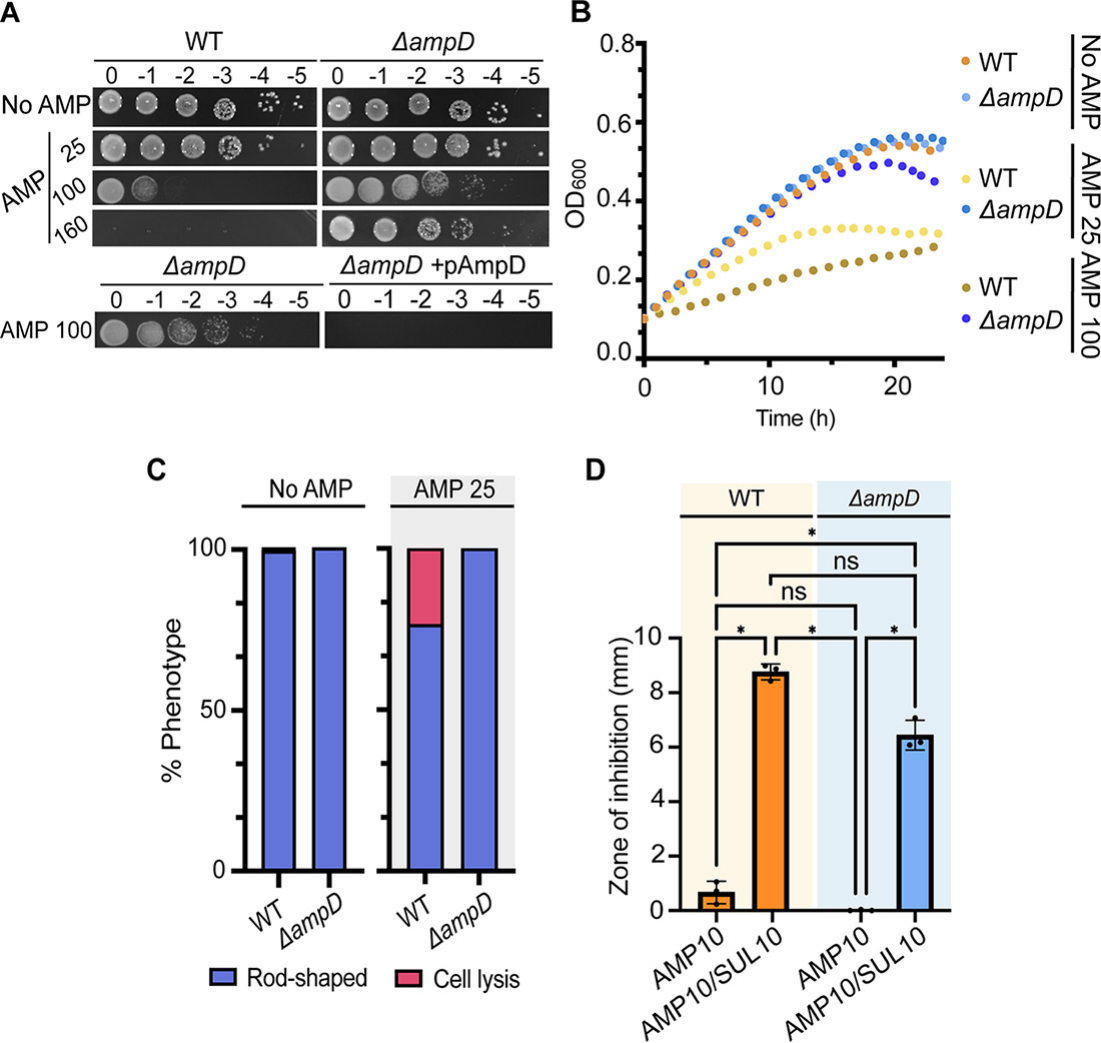
Loss of AmpD results in constitutive β-lactamase activity and elevated ampicillin resistance. (A) Ampicillin susceptibility assay performed by spotting dilutions. Briefly, exponential cultures were serially diluted and spotted on solid medium containing no ampicillin (No AMP) or ampicillin at 25, 100, or 160 μg/mL (AMP 25, AMP 100, or AMP 160, respectively) and incubated at 28°C for ~40 h before imaging. Plates used to demonstrate complementation of Δ*ampD* (Δ*ampD* + pAmpD) included 1 μM IPTG to induce expression of plasmid-encoded AmpD. (B) Growth of A. tumefaciens WT and Δ*ampD* in the absence (No AMP) and presence of various concentrations of ampicillin (AMP 25 or AMP 100) for 24 h (*n* = 1; 2 replicates). (C) Quantitative analysis of phase-contrast microscopy of exponentially growing strains treated with ampicillin at 25 μg/mL (AMP 25). The percent phenotype was calculated by counting the number of cells displaying one of the indicated phenotypes (1 cell = 1 phenotype) and dividing it by the total number of cells for each strain. (D) Disk susceptibility assay performed on a lawn of indicated strains grown on LB plates for 24 h at 28°C. AMP 10, disk containing 10 μg/mL ampicillin; AMP 10/SUL 10, disk containing 10 μg/mL ampicillin and 10 μg/mL sulbactam, a broad-spectrum β-lactamase inhibitor. Data represent the mean (±SD) of three independent experiments. ****, *P < *0.0001; ***, *P < *0.001; **, *P < *0.01; *, *P* < 0.1; ns, not significant.

Given that in A. tumefaciens ampicillin triggers the AmpC-dependent production of β-lactamases, we hypothesized that if AmpD was an anhydro amidase involved in the recycling of cell wall degradation fragments, its loss should result in increased AmpR-activating fragments in the cytoplasm, β-lactamase induction, and ampicillin resistance. First, we found that Δ*ampD* cells exhibit normal cell viability (Fig. 2A), cell growth (Fig. 2B), and morphology (Fig. S2B and C). Δ*ampD* cells are highly resistant to ampicillin (Fig. 2A). Indeed, WT cells spotted on AMP 160 are not viable, whereas Δ*ampD* cells spotted on AMP 160 display only an ~10-fold decrease in viability compared to that of untreated cells. In contrast, production of *ampD* from an IPTG (isopropyl-β-d-thiogalactopyranoside)-inducible plasmid (+pAmpD) resulted in a 100,000-fold decrease in viability in the presence of AMP 100 (Fig. 2A). In liquid, Δ*ampD* cells continue to grow normally, even in the presence of AMP 100 (Fig. 2B), and ampicillin treatment does not trigger obvious morphological changes or cell lysis (Fig. S2B and C; Fig. 2C). Δ*ampD* cells produce readily detectable amounts of β-lactamase in both the presence and absence of ampicillin (Fig. S2D). The increased zone of inhibition observed in the presence of ampicillin and sulbactam is consistent with the high level of ampicillin resistance observed in Δ*ampD* cells being mediated by a β-lactamase (Fig. 2D). Together, these results indicate that loss of AmpD leads to derepression and increased β-lactamase activity. Our findings are consistent with other bacterial models such as P. aeruginosa, where deletion of 1,6-anhydro amidases involved in the recycling of AmpR-activating ligands leads to increased β-lactamase expression (52–55) due to the buildup of activating ligands in the cytoplasm.

### AmpC is constitutively produced in Δ*ampD* cells.

**FIG 3:**
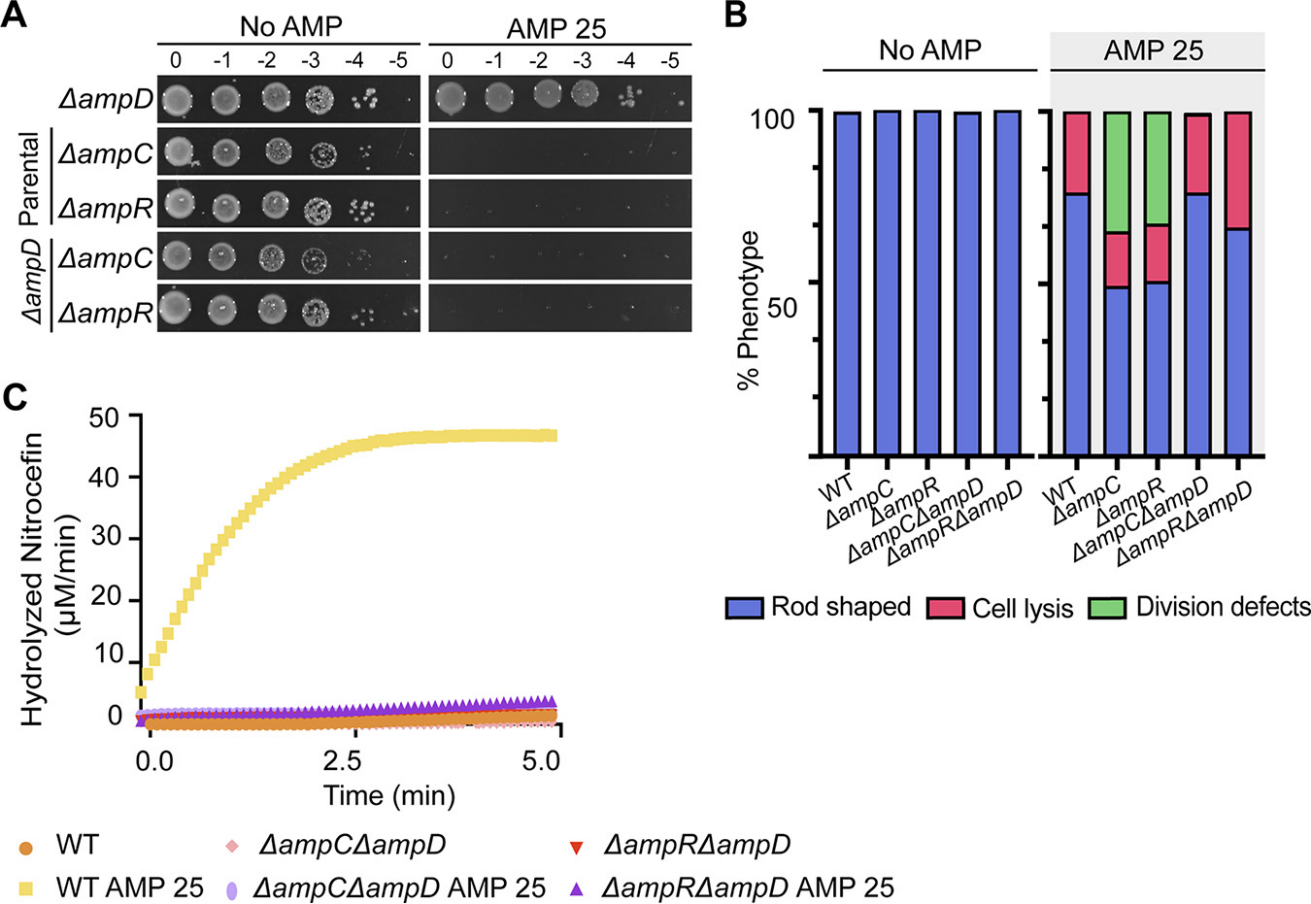
AmpC is the β-lactamase that confers Δ*ampD* cells elevated ampicillin resistance. (A) An ampicillin susceptibility assay was performed by spotting dilutions. Briefly, exponentially grown cultures were serially diluted, spotted on solid medium containing no ampicillin (No AMP) or ampicillin at 25 μg/mL (AMP 25), and incubated at 28°C for ~40 h before imaging. (B) Quantitative analysis of phase-contrast microscopy of exponentially growing strains untreated (No AMP) or treated with ampicillin at 25 μg/mL (AMP 25). The percent phenotype was calculated by counting the number of cells displaying one of the indicated phenotypes (1 cell = 1 phenotype) and dividing it by the total number of cells for each strain. (C) Determination of β-lactamase production was performed by a nitrocefin assay using cell lysates. No AMP or AMP 25 indicates cells untreated or treated with ampicillin at 25 μg/mL, respectively, for 2 h before the generation of cell lysates. Data represent one of two biological replicates.

We have shown that AmpC and AmpR are required for ampicillin resistance (Fig. 1) and that loss of AmpD leads to elevated β-lactamase activity and ampicillin resistance in A. tumefaciens C58 (Fig. 2). To confirm that AmpC is the β-lactamase produced by the Δ*ampD* strain, we deleted *ampC* or *ampR* in the Δ*ampD* background (Fig. 3). In the absence of ampicillin, we found that Δ*ampC* Δ*ampD* and Δ*ampR* Δ*ampD* cells display normal cell viability, growth, and morphology (Fig. 3A and B; Fig. S3A and B). However, we found that treatment with AMP 25, on either solid medium or liquid medium, is lethal to Δ*ampC* Δ*ampD* or Δ*ampR* Δ*ampD* cells (Fig. 3A; Fig. S3A). Treatment of Δ*ampC* Δ*ampD* or Δ*ampR* Δ*ampD* cells with AMP 25 for 2 h results in lysis of 22.9% and 33.8% of the cells, respectively, in comparison to untreated cells (No AMP), where lysis is not readily observed (Fig. 3B; Fig. S3B). In comparison to Δ*ampC* or Δ*ampR* cells, where cell division defects are observed in >20% of the population, very few Δ*ampC* Δ*ampD* or Δ*ampR* Δ*ampD* cells exhibit cell division defects when treated with AMP 25 (Fig. 3B). The low incidence of cell division defects observed in Δ*ampC* Δ*ampD* or Δ*ampR* Δ*ampD* cells suggests that the activity of AmpD contributes to the inefficient cell division of Δ*ampC* and Δ*ampR* cells following ampicillin treatment. Finally, to assess whether the Δ*ampD* strain could induce β-lactamase production in the absence of *ampC* or *ampR*, we performed nitrocefin assays. Δ*ampC* Δ*ampD* and Δ*ampR* Δ*ampD* cells fail to produce detectable levels of β-lactamase in the absence or presence of AMP 25 (Fig. 3C). Together, these results suggest that induction of AmpC is the main cause for the elevated resistance to ampicillin observed in Δ*ampD* cells. These data are consistent with the current model for β-lactam resistance in P. aeruginosa, where the loss of anhydro amidases leads to accumulation of cell wall degradation products that activate AmpR, leading to derepression of AmpC (53–55, 68).

### Absence of MltB3 (Δ*atu3779*) leads to a failure of AmpC-dependent induction of β-lactamases.

Lytic transglycosylases (LTs) are likely to function as the enzymes that generate the AmpR-activating fragments. Different families of LTs have been linked to β-lactam resistance in several bacterial organisms (78, 79). For instance, in Caulobacter crescentus, deletion of *sdpA*, which encodes a soluble LT, led to increased sensitivity to ampicillin (80). In P. aeruginosa, loss of several *mltBs* and/or *slt* led to a decrease in the β-lactam MIC, cell viability, and increased outer membrane permeability (81, 82). Thus, we sought to determine if LTs contribute to the β-lactam resistance of A. tumefaciens. The A. tumefaciens genome encodes 8 putative LTs belonging to 3 families: family 1, the soluble lytic transglycosylases (Slt); family 2, membrane-bound lytic transglycosylase A (MltA); and family 3, membrane-bound lytic transglycosylase B (MltB) (Fig. S4A). We found that single deletions of LTs did not affect cell viability, suggesting a wide redundancy of functions between LTs (Fig. S4B).

**FIG 4:**
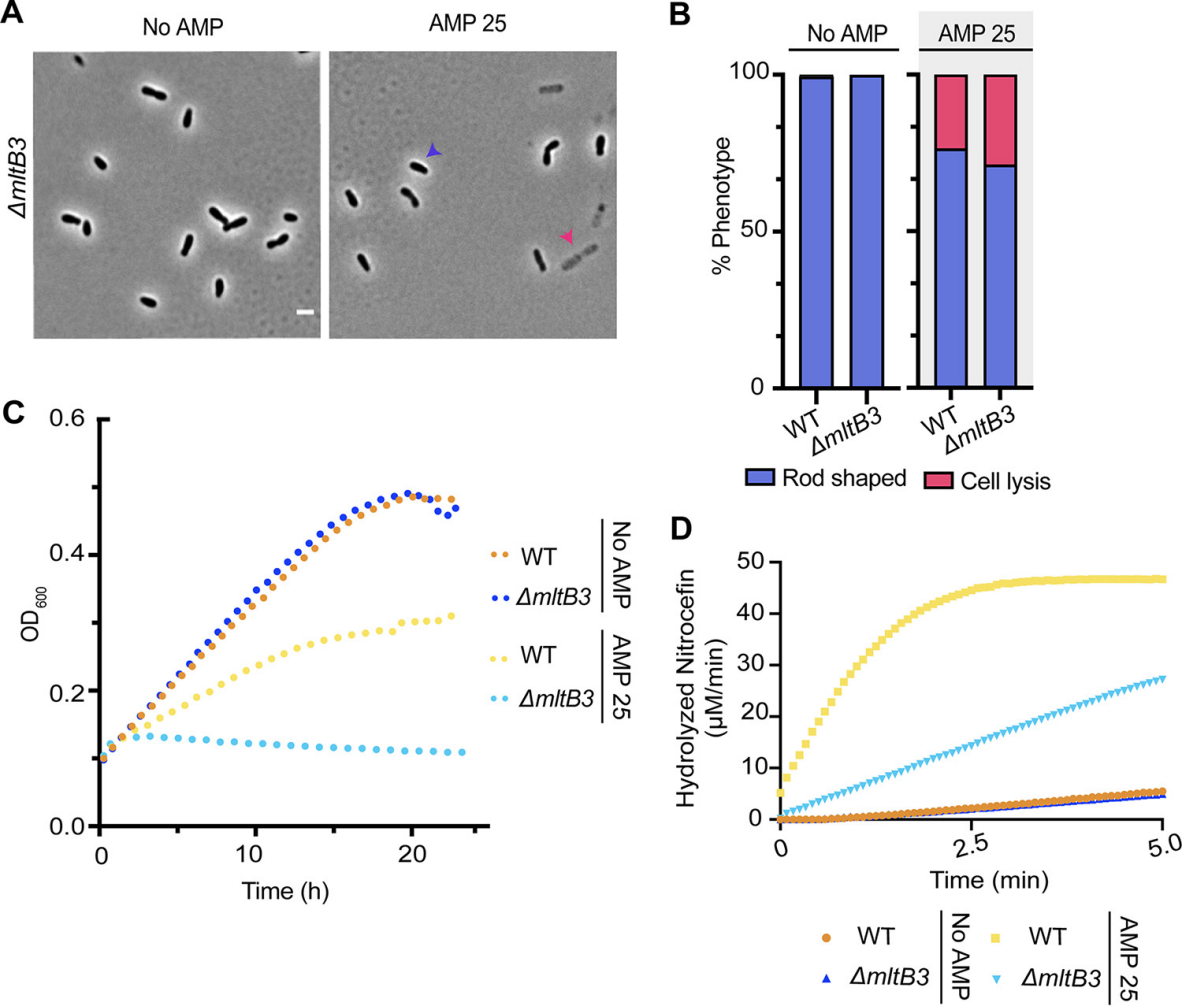
MtlB3 is required for ampicillin resistance in A. tumefaciens. (A) Phase-contrast microscopy of exponentially growing strains treated with ampicillin at 25 μg/mL (AMP 25) for 2 h. (B) Quantitative analysis of phase-contrast microscopy of exponentially growing strains treated with ampicillin at 25 μg/mL (AMP 25) for 2 h. The percent phenotype was calculated by counting the number of cells displaying one of the phenotypes indicated (1 cell = 1 phenotype) and dividing it by the total number of cells for each strain. (C) Growth of A. tumefaciens WT and Δ*mltB3* in the absence (No AMP) and presence of ampicillin at 25 μg/mL (AMP 25) for 24 h (*n* = 1; 2 replicates). (D) Determination of β-lactamase production was performed by a nitrocefin assay using cell lysates. No AMP or AMP 25 indicates cells untreated or treated with ampicillin at 25 μg/mL, respectively, for 2 h before the generation of cell lysates. Data shown represent one of two biological replicates.

Despite the potential for functional redundancy, we found that deletion of a single, family 3, membrane-bound lytic transglycosylase, MltB3 (*atu3779*), causes ampicillin hypersensitivity (Fig. S4B). Treatment of Δ*mltB3* cells with AMP 25 for 2 h causes a cell lysis defect (28.8%) (Fig. 4A and B) and results in a severe growth defect (Fig. 4C), indicating that MltB3 is required for ampicillin resistance. Production of MltB3 from an IPTG-inducible plasmid (+pMltB3) in Δ*mltB3* cells restores viability in the presence of AMP 25 (Fig. S4B), confirming that MltB is responsible for this phenotype. Finally, Δ*mltB3* cells exhibit reduced production of β-lactamase after AMP 25 treatment for 2 h (Fig. 4D). Together, these results suggest that in A. tumefaciens, MltB3 is a specialized enzyme that functions in the AmpR-AmpC β-lactamase pathway. These observations contrast with the P. aeruginosa model, in which the β-lactam sensitivity of LT mutants is due to increased outer membrane permeability rather than β-lactamase production (81).

### Plant transformation with Δ*mltb3* cells requires a low concentration of ampicillin for the elimination of bacteria.

**FIG 5:**
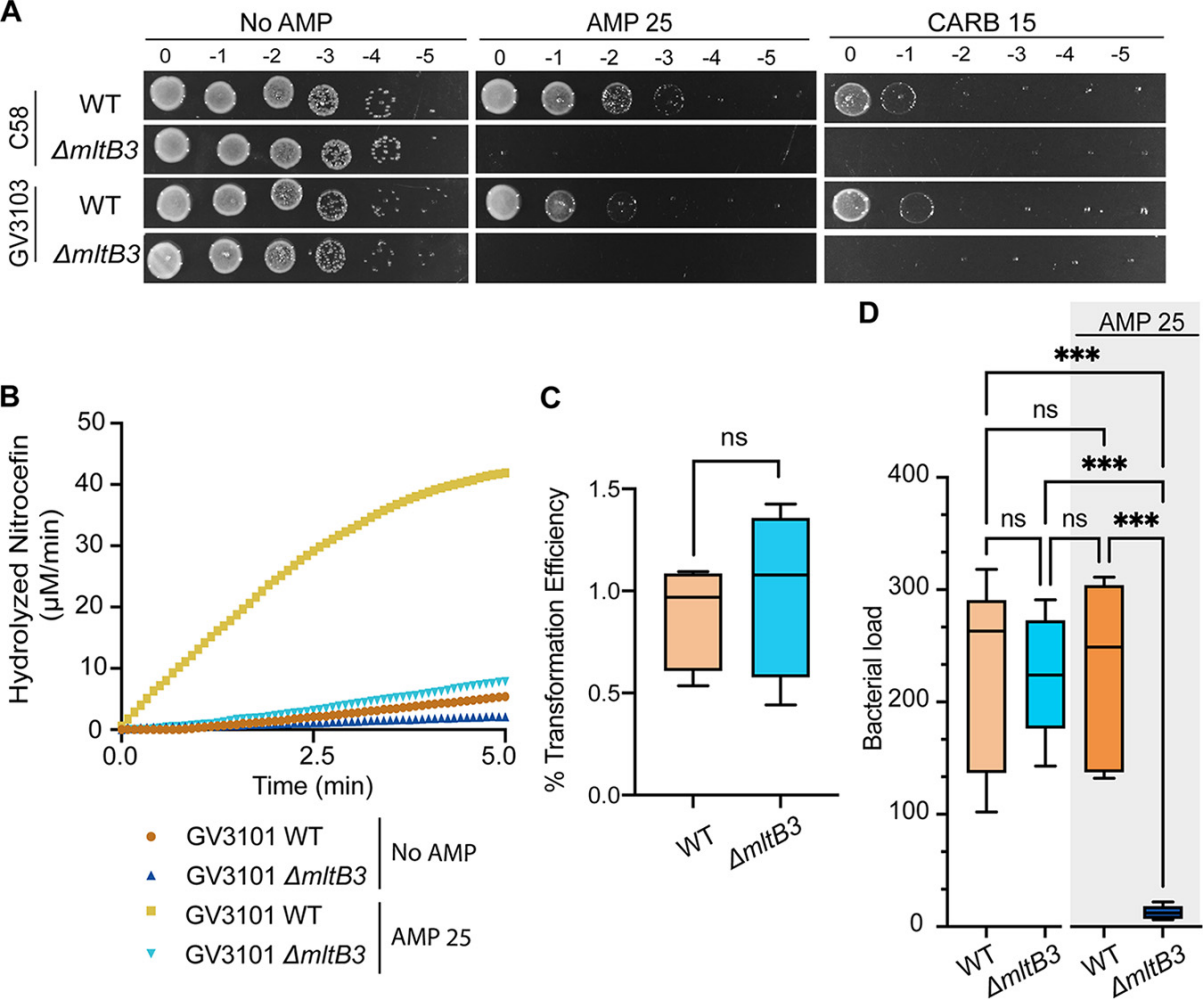
A. tumefaciens GV3103 Δ*mltB3* can be used to transform plants efficiently, and bacteria can be removed using low concentrations of ampicillin. (A) An ampicillin susceptibility assay was performed by spotting dilutions. Briefly, exponentially grown cultures were serially diluted, spotted on solid medium containing no ampicillin (No AMP), ampicillin at 25 μg/mL (AMP 25), or carbenicillin at 15 μg/mL (CARB 15), and incubated at 28°C for ~40 h before imaging. (B) Determination of β-lactamase production was performed by a nitrocefin assay using cell lysates. Data shown represent one of two biological replicates. No AMP or AMP 25 indicates cells untreated or treated with ampicillin at 25 μg/mL, respectively, for 2 h before the generation of cell lysates. (C and D) Transformation efficiency (C) and bacterial loads (D) of seeds transformed with WT A. tumefaciens GV3101 and GV3101 Δ*mltB3* using the floral dip assay technique (83).

Next, we sought to determine if the ampicillin-sensitive strains of A. tumefaciens constructed in this work are competent for plant transformation. While the Δ*ampC* strain appears to be the ideal mutant for these studies, we considered the impact of the mutation on the overall fitness of our ampicillin-sensitive strains. The growth dynamics of Δ*ampC* and Δ*ampR* strains are very similar to those of the WT in liquid medium; however, these strains exhibit a ~10-fold viability defect on solid medium. In contrast, Δ*mltB3* cell growth dynamics mimic those of the WT and lyse quickly in the presence of low concentrations of ampicillin. Thus, we reasoned that the Δ*mltB3* allele would enable us to test the transformation efficiency of an otherwise fit but ampicillin-sensitive A. tumefaciens strain. To this end, we deleted *mltB3* in A. tumefaciens GV3103 and found that this mutation causes susceptibility to AMP 25 and carbenicillin at 15 μg/mL (CARB 15) (Fig. 5A). To confirm that the absence of MltB3 (Δ*mltB3*) prevented the induction of β-lactamase production in the GV3101 strain after ampicillin treatment, we performed nitrocefin assays using lysates of cells treated with AMP 25 for 2 h. While β-lactamase activity was readily detected in WT GV3101 cells treated with AMP 25, the Δ*mltB3* mutant produced relatively low levels of inducible β-lactamase (Fig. 5B). Together, we conclude that MltB3 is the major LT in A. tumefaciens C58 and GV3101 contributing to the natural resistance of A. tumefaciens to ampicillin and other β-lactam antibiotics.

Next, we confirmed that Δ*mltB3* A. tumefaciens GV3101 effectively transforms Arabidopsis thaliana by use of the standard floral dip technique (83) with an efficiency comparable to that of the WT (Fig. 5C). We then asked whether elimination of the bacteria could be achieved by using low concentrations of ampicillin (AMP 25). Seeds transformed with WT A. tumefaciens GV3101 contained similar bacterial loads as seeds transformed with Δ*mltb3* A. tumefaciens GV3101 when plated on solid medium without ampicillin (Fig. 5D). When plated on medium containing AMP 25, seeds transformed with Δ*mltb3* GV301 exhibited a significant drop in bacterial load. In contrast, this low level of ampicillin did not reduce the bacterial load of seeds transformed with WT GV3101 (Fig. 5D). These results demonstrate that the Δ*mltb3* GV3101 strain is useful for the transformation of Arabidopsis thaliana and that elimination of the bacteria can be achieved by using lower concentrations of ampicillin. While ampicillin or carbenicillin is occasionally used for clearing *Agrobacterium* after transformation, many labs routinely use expensive antibiotics such as the proprietary blends of ticarcillin-clavulanate (Timentin) and amoxicillin-clavulanate (Augmentin) (84, 85). Our work highlights the ability to clear Δ*mltB* cells using ampicillin, a cost-effective and readily available antibiotic. Furthermore, the increased sensitivity of this strain to carbenicillin suggests that bacterial clearance following plant transformation can likely be achieved by using other β-lactam antibiotics. Overall, these data show the potential impact of improved understanding of the cell biology of A. tumefaciens to improve genetic engineering approaches.

### Concluding remarks and future perspectives.

The natural ability of A. tumefaciens to transform plants has allowed the production of transgenic crops of incredible economic importance for the past 4 decades. One challenge of the A. tumefaciens-mediated plant transformation is the natural resistance of A. tumefaciens to antibiotics, which requires toxic concentrations of antibiotics to eliminate A. tumefaciens from transformed tissues. Here, we show that A. tumefaciens induces β-lactamase activity in response to ampicillin exposure. Indeed, induction of β-lactamase activity upon exposure to ampicillin is dependent on the β-lactamase AmpC and the transcription factor AmpR. Moreover, we found that deletion of a single LT, MltB3, sensitizes A. tumefaciens to the β-lactams.

**FIG 6:**
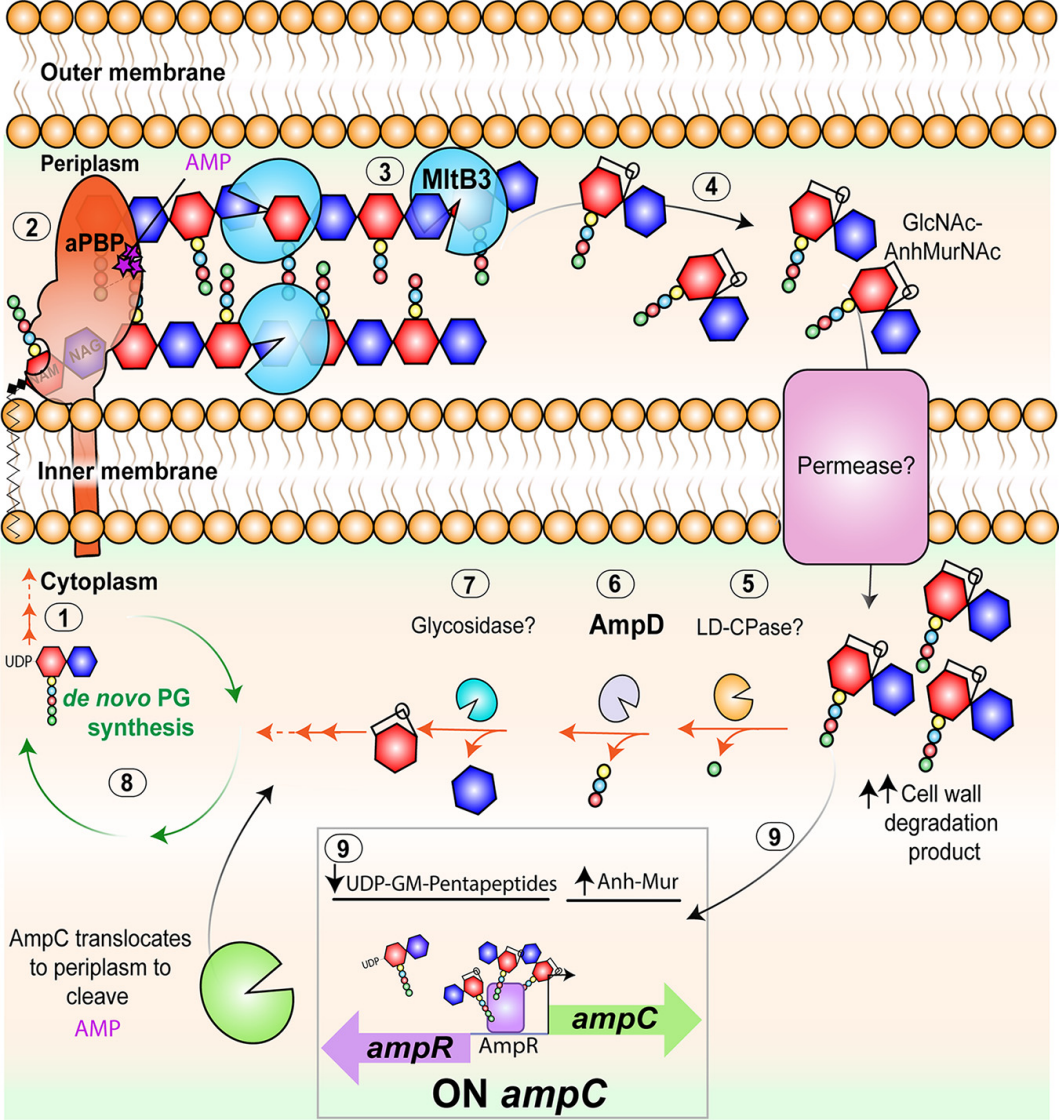
Working model for A. tumefaciens ampicillin resistance. Bifunctional PBPs extend the cell wall through the transglycosylation (linking of carbohydrates) and transpeptidation (linking of peptide stems) reactions using cytoplasmic precursors (step 1). β-Lactams such as ampicillin (purple stars) target the transpeptidase domain of penicillin-binding proteins (PBPs) (step 2), leading to a block in bacterial cell growth and increased hydrolytic activity by lytic transglycosylases. In A. tumefaciens, inactivation of the lytic transglycosylase MltB3 results in inhibition of β-lactamase derepression and lysis, suggesting that MltB3 is likely required for the generation of cell wall degradation products (step 3) that are transported to the cytoplasm (step 4). In the cytoplasm, hydrolytic enzymes (steps 5 to 7) digest cell wall degradation products and promote PG recycling, enabling *de novo* PG synthesis (step 8). Similar to treatment with β-lactams, where a block in cell growth leads to an increase in cell wall degradation products (Anh-Mur), inactivation of anhydro amidases such as AmpD (step 6) increases the pool of cell wall degradation products, leading to β-lactam resistance. In A. tumefaciens, inactivation of AmpD leads to derepression of β-lactamases and ampicillin resistance (step 9). Both AmpC, an inducible β-lactamase that is under the transcriptional control of AmpR, and AmpR seem to be responsible for the derepression observed in Δ*ampD* cells. Thus, our working model suggests that upon ampicillin exposure, a block in growth leads to increased activity of MltB3. An increase in cell wall degradation products leads to induction of AmpC expression by AmpR and the presumed translocation of AmpC to the periplasm, resulting in ampicillin resistance.

We propose that during A. tumefaciens growth and remodeling, there is a delicate balance between the synthesis and degradation of the bacterial cell wall. PBPs insert precursor cytoplasmic monomers into the growing cell wall polymer (Fig. 6, steps 1 and 2). During remodeling, cell wall hydrolytic enzymes such as endopeptidases and LTs, including MltB3, liberate cell wall degradation products, which are transported back into the cytoplasm of A. tumefaciens for their recycling (Fig. 6, steps 3 and 4). In the cytoplasm, hydrolytic enzymes, including l,d-carboxypeptidases (LD-CPases), amidases such as AmpD, and glycosidases, limit the pool of cell wall fragments by allowing their recycling (Fig. 6, steps 5 to 8). When cell wall degradation fragments accumulate during β-lactam treatment, the AmpR-dependent production of the AmpC β-lactamase is increased (Fig. 6, step 9). Overall, the identification and contributions of genes conferring ampicillin resistance in A. tumefaciens will be beneficial for improving the design of A. tumefaciens*-*mediated genetic engineering.

## MATERIALS AND METHODS

### Bacterial strains and growth conditions.

Unless otherwise indicated, Agrobacterium tumefaciens GV3101, C58, and derived strains were grown in LB medium (10 g/L tryptone, 5 g/L yeast extract, and 10 g/L NaCl) at 28°C with shaking, and the antibiotic kanamycin was added at a concentration of 100 μg/mL (KAN 100) to maintain plasmids in complementing strains. For determining bacterial loads after transformation, Agrobacterium tumefaciens GV3101 was grown in AT minimal medium with glucose and (NH_4_)_2_SO_4_ (ATGN) (86). E. coli DH5α and S17.1 were routinely cultivated in LB medium at 37°C with shaking, and the antibiotic kanamycin was added at concentrations of 50 μg/mL and 25 μg/mL to maintain plasmids in the DH5α and S17.1 strains, respectively.

### Construction of plasmids and strains.

All strains and plasmids used are listed in Table 1. Synthesized DNA primers are listed in Table 2.

**TABLE 1:** Strains and plasmids used in this study

Strain or plasmid	Relevant characteristics	Reference/source
Source plasmids		
pSRKKm-Plac-sf*gfp*	pSRKKm vector containing *lacI*^q^ and *lac* promoter with sfGFP	(90)
pNTPS139	Km^r^; suicide vector containing *oriT* and *sacB*	D. Alley
		
Deletion plasmids		
pNTPS139 Δ*atu3077*	Km^r^ Suc^s^; deletion plasmid for *atu3077* (*ampC*)	This study
pNTPS139 Δ*atu3078*	Km^r^ Suc^s^; deletion plasmid for *atu3078* (*ampR*)	This study
pNTPS139 Δ*atu0009*	Km^r^ Suc^s^; deletion plasmid for *atu0009* (*mltA*)	This study
pNTPS139 Δ*atu0092*	Km^r^ Suc^s^; deletion plasmid for *atu0092* (*mltB1*)	This study
pNTPS139 Δ*atu2122*	Km^r^ Suc^s^; deletion plasmid for *atu2122* (*mltB2*)	This study
pNTPS139 Δ*atu3779*	Km^r^ Suc^s^; deletion plasmid for *atu3779* (*mltB3*)	This study
pNTPS139 Δ*atu1022*	Km^r^ Suc^s^; deletion plasmid for *atu1022* (*slt1*)	This study
pNTPS139 Δ*atu2122*	Km^r^ Suc^s^; deletion plasmid for *atu2112* (*slt2*)	This study
pNTPS139 Δ*atu2117*	Km^r^ Suc^s^; deletion plasmid for *atu2117* (*slt3*)	This study
pNTPS139 Δ*atu3093*	Km^r^ Suc^s^; deletion plasmid for *atu3093* (*slt4*)	This study
pNTPS139 Δ*atu2113*	Km^r^ Suc^s^; deletion plasmid for *atu2113* (*ampD*)	This study
		
Replicating plasmid*s*		
Plac-*atu3077*	Km^r^; pSRKKm vector containing *lacI*^q^ and *lac* promoter for the production of AmpC	This study
Plac-*atu3078*	Km^r^; pSRKKm vector containing *lacI*^q^ and *lac* promoter for the production of AmpR	This study
Plac-*atu2113*	Km^r^; pSRKKm vector containing *lacI*^q^ and *lac* promoter for the production of AmpD	This study
Plac-*atu3779*	Km^r^; pSRKKm vector containing *lacI*^q^ and *lac* promoter for the production of MltB3	This study
		
Strains		
E. coli		
DH5α	Cloning strain	Life Technologies
S17.1	Sm^r^; RP4-2, Tc::Mu,Km-Tn*7*, for plasmid mobilization	(91)
A. tumefaciens		
C58	Nopaline type strain; pTiC58; pAtC58	
GV3101	C58-derived; pTiC58DT-DNA; strain for *Agrobacterium*-mediated transformation of dicots	John Walker lab
FC2452	Deletion strain for Δ*mltA* (*atu0009*) in C58	This study
FC2444	Deletion strain for Δ*mltB1* (*atu0092*) in C58	This study
FC2465	Deletion strain for Δ*mltB2* (*atu2122*) in C58	This study
FC2487	Deletion strain for Δ*mltB3* (*atu3779*) in C58	This study
FC2446	Deletion strain for Δ*slt1* (*atu1022*) in C58	This study
FC2448	Deletion strain for Δ*slt2* (*atu2112*) in C58	This study
FC2450	Deletion strain for Δ*slt3* (*atu2117*) in C58	This study
C58 Δ*tetRA*::a-*att*Tn*7*	Replacement of the Δ*tetRA* locus with an artificial *att*Tn*7* site	(90)
C58 Δ*tetRA*::a-*att*Tn*7* Δ*atu3077*	Deletion strain for Δ*ampC*	This study
C58 Δ*tetRA*::a-*att*Tn*7* Δ*atu3077* pSRKKm-Plac-*atu3077*	Km^r^; deletion of *ampC* in C58Δ*tetRA*::a-*att*Tn*7* carrying complementing plasmid	This study
C58 Δ*tetRA*::a-*att*Tn*7* Δ*atu3078*	Deletion strain for Δ*ampR*	This study
C58 Δ*tetRA*::a-*att*Tn*7* Δ*atu3078* pSRKKm-Plac-*atu3078*	Km^r^; deletion of *ampR* in C58Δ*tetRA*::a-*att*Tn*7* carrying complementing plasmid	This study
C58 Δ*tetRA*::a-*att*Tn*7* Δ*ampD*	Deletion strain for Δ*ampD*	This study
C58 Δ*tetRA*::a-*att*Tn*7* Δ*ampD* pSRKKM-Plac-*atu2113*	Km^r^; deletion of *ampD* in C58Δ*tetRA*::a-*att*Tn*7* carrying complementing plasmid	This study
C58 Δ*tetRA*::a-*att*Tn*7* Δ*ampD* Δ*ampC*	Deletion strain for Δ*ampD* Δ*ampC*	This study
C58 Δ*tetRA*::a-*att*Tn*7* Δ*ampD* Δ*ampR*	Deletion strain for Δ*ampD* Δ*ampR*	This study
C58 Δ*tetRA*::a-*att*Tn7 Δ*atu*3093	Deletion strain for Δ*slt*4	This study
C58 GV3101 Δ*mltB3*	Deletion strain for Δ*mltB3*	This study

**TABLE 2:** Synthesized DNA primers used in this study

Synthesized DNA^*a*^	Sequence
*atu3077* P1	5′-GCGGCGACTAGTAAACGGATGCCGCTTTTGAAATGC-3′
*atu3077* P2	5′-AAGCTTGGTACCGAATTCGCGATTAAATTTCATCTTTCGTGT-3′
*atu3077* P3	5′-GAATTCGGTACCAAGCTTGCGCTCGAAAAGGCGCAATAA-3′
*atu3077* P4	5′-GTCGTCGGATCCAGATAACTCGGCACACGCCCA-3′
*atu3077* P5	5′-CTGCGCCGCCGGTGAAACGCCCGC-3′
*atu3077* P6	5′-TGTGCCGGAGGCGCTTGCGATCGC-3′
*atu3078* P1	5′-GTCGTCACTAGTGGGTTTTCCCTTCATGGACGG-3′
*atu3078* P2	5′-AAGCTTGGTACCGAATTCAAATTGCCGAACCATTCAAGACCT-3′
*atu3078* P3	5′-GAATTCGGTACCAAGCTTGAGACCATCGGAACGGCGTGA-3′
*atu3078* P4	5′-GTCGTCGGATCCCCTGTTTGATGCTTTTTATCGCGC-3′
*atu3078* P5	5′-CCAAGCGCCGGGAAAAGCGTGTCC-3′
*atu3078* P6	5′-GGCATCGTCTGCGTGGTGTTCGTC-3′
*atu2113* P1	5′-GTCGTCACTAGTCAGATTTTCGATTTCCCGGACGAA-3′
*atu2113* P2	5′-AAGCTTGGTACCGAATTCCGAACATTCTTTCATGCGACG-3′
*atu2113* P3	5′-GAATTCGGTACCAAGCTTCTGCCGAGATTTTCGGCCGCCTGA-3′
*atu2113* P4	5′-GTCGTCGGTACCCCACCGAAACCACGGCATGCGCCA-3′
*atu2113* P5	5′-GCGCAATCGGCGACGCGG-3′
*atu2113* P6	5′-GTCGTCTGCTGCACACTTCGCCGC-3′
*atu0009* P1	5′-AAAAAGCTTAACGCATCTTCTAGCCTTGCG-3′
*atu0009* P2	5′-TATTCATTGCTCGGATTCGG-3′
*atu0009* P3	5′-GCAATGAATATATCGGCGATGAAAGGC-3′
*atu0009* P4	5′-AAAGGATCCGAAAGAACAATTCCTCCGC -3′
*atu0009* P5	5′-TTTCGGCGACCTATGACAAGGACGG-3′
*atu0009* P6	5′-TTACCAGTTTGCGGAACGCTGGG-3′
*atu0092* P1	5′-AAACTGCAGATTCTTGCCCTGATGCCCATTGTCGC-3′
*atu0092* P2	5′-AGACCGAATATCGTCTTTTAATGCTGGTCGG-3′
*atu0092* P3	5′-ATTCGGTCTCCTCTTGGATGG-3′
*atu0092* P4	5′-AAAGGATCCAAGTCGAGATCGACTGAGCCC-3′
*atu0092* P5	5′-AATATGTCCGCCACAACCATCGTCGC-3′
*atu0092* P6	5′-ATTACATCACAGACCGCCTCTCCG-3′
*atu2122* P1	5′-AAAGAATTCAATCATCAGGGTTCCAATGCGG-3′
*atu2122* P2	5′-TAGTGCGATTTTCCTCGATAGGTTGTTGGC-3′
*atu2122* P3	5′-ATCGCACTACCGGGCCTTTAATCTATCGG-3′
*atu2122* P4	5′-AAAAAGCTTATAATGACGTCTTTGAACGC-3′
*atu2122* P5	5′-TATACCGCAACCGGCGTCGTACCCG-3′
*atu2122* P6	5′-TTTCTGTGATGCGGTGCAGCACGG-3′
*atu3779* P1	5′-AAACTGCAGTAGAAATTCGACGGCGCCG-3′
*atu3779* P2	5′-TTTCGATTGCGAAAACGCATCGGGCG-3′
*atu3779* P3	5′-GCAATCGAAATAATGTGCCGGCGAATTCGG-3′
*atu3779* P4	5′-AAAGGATCCTTGGCCGTTCATGTCGTAGCC-3′
*atu3779* P5	5′-TTTCGGAACTGCCTTGGTGGCGG-3′
*atu3779* P6	5′-ATTCCCGGCCGGAACTACCATCGC-3′
*atu2112* P1	5′- AAAAAGCTTTATTTCGTCTTCGAGGATGGG-3′
*atu2112* P2	5′- GATGATCGATATGACAGAAACAGTGAAATGGC-3′
*atu2112* P3	5′-ATCGATCATCTGCGAAATTGCG-3′
*atu2112* P4	5′-AAAGAATTCATATCGTCCTCGGTTTCCGC-3′
*atu2112* P5	5′- AAAAAGCTTTATTTCGTCTTCGAGGATGGG-3′
*atu2112* P6	5′- TTTGCACCGAAAGATGCCGCG-3′
*atu1022* P1	5′-AAAAAGCTTTTATGCGCTTTGACCAGCGCACCC-3′
*atu1022* P2	5′-AATCCCCAGACTGTCTTTTTCATGCCG-3′
*atu1022* P3	5′-TGGGGATTATTCAGGCACGGGCTAGCC-3′
*atu1022* P4	5′-AAAGAATTCAATACGCTCTTCAACTCCATCCG-3′
*atu1022* P5	5′- AAAAGCGACGTCGCTCGCG-3′
*atu1022* P6	5′-AAACTACTACGACGAAGACGGTCAGG-3′
*atu2117* P1	5′-AAAGAATTCAAGAGAATGTCTGGACAGGCGTGGC-3′
*atu2117* P2	5′-ATTTTCAATCTAGAGTTGCCGCCTTCGTTATGCC-3′
*atu2117* P3	5′- TTGAAAATGCTGCGGCTCCC-3′
*atu2117* P4	5′-AAAAAGCTTTAGATGTTCCTGTCGAACACCG-3′
*atu2117* P5	5′-ATACAGGCGCATCGCGGCC-3′
*atu2117* P6	5′-AAGGCGAGGTGGTCACTGACC -3′
*atu3093* P1	5′-GCTGCAACTAGTGCGCCACAGCCCATCTCG-3′
*atu3093* P2	5′-AAGCATGGTACCGAATTCGCGGCTTGTTGATATTCCT-3′
*atu3093* P3	5′-GAATTCGGTACCATGCTTTAGCAGCGGCGGGCGCCGGA-3′
*atu3093* P4	5′-GCACGTAAGCTTGCGGTGATCTTGATGAT-3′
*atu3093* P5	5′-GAGCTTCTCGGGCAATTCCG-3′
*atu3093* P6	5′-GCCTCACGAAGCCGCACGATC-3′
*atu3077* NdeI Fwd	5′- GCGGCGCATATGAAATTTAATCGCAGACAT-3′
*atu3077* NdeI BamHI Rvs	5′- GTCGTCGGATCCTTATTGCGCCTTTTCGAG-3′
*atu3078* NdeI Fwd	5′- GCGGCGCATATGGTTCGGCAATTTCTTCCC-3′
*atu3078* BamHI Rvs	5′- GTCGTCGGATCCTCACGCCGTTCCGATGGT-3′
*atu3779* Ndel Fwd	5′- GCGGCGCATATGACAAAGACCCTTTCAAAT-3′
*atu3779* KpnI Rvs	5′- GCGGCGCATATGACAAAGACCCTTTCAAAT-3′
*atu2113* NdeI Fwd	5′- GCGGCGCATATGAAAGAATGTCTGCCGGAT-3′
*atu2113* BamHI Rvs	5′- GTCGTCGGATCCTCAGGCGGCCGAAAATCT-3′

a Fwd, forward; Rvs, reverse.

Gene deletions were achieved by allelic exchange, and vectors were constructed as previously described (87). Briefly, 500-bp fragments upstream and downstream of the gene of interest were amplified from purified C58 genomic DNA using primer pair P1/P2, which amplifies 500 bp upstream of the gene of interest, and primer pair P3/P4, which amplifies 500 bp downstream of the gene of interest. Overlapping PCR was used to merge and amplify the amplicons generated by P1/P2 and P3/P4, using primer pair P1/P4. The 1,000-bp amplicon was digested and ligated into a deletion plasmid, pNTPS139. The deletion plasmids were introduced into A. tumefaciens by mating using an E. coli S17.1 conjugation strain to create kanamycin (KAN)-resistant, sucrose-sensitive primary exconjugants. Deletion strains were constructed as described previously (73). Briefly, primary exconjugants were grown overnight at 28°C in ATGN with no selection and plated in ATGN plus KAN 300 for 48 h at 28°C. Colonies were screened by patching for KAN resistance and sucrose sensitivity. Colony PCR using primers P5/P4 was used to confirm that recombination took place and at the region of interest. Next, positive colonies are grown in ATGN at 28°C overnight and plated on AT minimal medium with sucrose and (NH_4_)_2_SO_4_ (ATSN). Secondary recombinants were screened by patching for sucrose resistance and KAN sensitivity. Colony PCR with primers P5/P6 for the respective gene target was used to confirm deletion. PCR products from P5/P6 primer sets were sequenced to further confirm deletions.

For the construction of replicating plasmids, the amplicons and pSRKKM-Plac-*sfgfp* were digested overnight, ligated overnight at 4°C using NEB T4 DNA ligase, and transformed into E. coli DH5α. Amplicons contain a stop codon and do not produce translational fusions to superfolder green fluorescent protein (sfGFP). Plasmids were sequenced to verify content and were introduced into A. tumefaciens by mating using E. coli S17.1 harboring the appropriate plasmid.

### Cell viability spot assays.

The cell viability assay was performed as described previously (88). For cell viability spot assays, cultures were grown overnight, diluted to an optical density at 600 nm (OD_600_) of 0.05, grown to exponential phase (OD_600_ of 0.4 to 0.6), and serially diluted in LB up to 10^−5^. Four microliters of each dilution was spotted onto LB plates and incubated at 28°C for 36 to 40 h before imaging. To determine ampicillin antibiotic resistance, A. tumefaciens cultures were grown overnight, diluted to an OD_600_ of 0.05, grown to exponential phase (OD_600_ of 0.4 to 0.6), serially diluted, and spotted onto LB plates containing AMP 25, AMP 100, or AMP 160. Similarly, IPTG-inducible complementing strains were grown overnight in the absence of IPTG, diluted to an OD_600_ of 0.05, grown to exponential phase (OD_600_ of 0.4 to 0.6), serially diluted, and spotted onto LB plates containing 1 μM IPTG, KAN 150, and AMP 25 or AMP 100.

### Phase-contrast microscopy.

For phase-contrast microscopy, 0.8 μL of exponentially grown cultures (OD_600_ of 0.4 of 0.6) was spotted onto a 1.25% agarose pad as previously described (88) using a Nikon Eclipse Ti inverted microscope and imaged using a Nikon Plan 60× oil Ph3 objective. Cell length analysis was performed using the MicrobeJ plug-in for Fiji (89). A one-way analysis of variance (ANOVA) Kruskal-Wallis test with Dunn’s posttest was used to compare the indicated strains. Images were prepared using Adobe Photoshop 2021, Adobe Illustrator 2021, and Prism 9.

### Growth curves.

For growth curves, exponentially growing cultures (OD_600_ of 0.4 to 0.6) were diluted to an OD_600_ of 0.2 and 100 μL of diluted culture was added to wells of a 96-well plate. OD_600_ readings were recorded using a plate reader at 28°C with shaking every 5 to 10 min. When indicated, ampicillin was added to a final concentration of 25 or 100 μg/mL (AMP 25 or AMP 100). Plots of OD_600_ data represent two technical replicates for each culture measured every 5 min for 24 to 48 h.

### Disk diffusion assay.

The disk diffusion assay was used to determine the resistance of *Agrobacterium* to various antibiotics. Cells were grown on LB agar plates. Sterile disks (6.5 mm in diameter) were placed on the surface of LB agar plates seeded with overnight cultures of the indicated strains. We used three different Thermo Scientific Oxoid antimicrobial susceptibility disks: blank (sterile disk containing no antibiotic), AMP 10 (ampicillin, 10 μg/mL), and AMP 10/SUL 10 (ampicillin, 10 μg/mL, plus sulbactam, 10 μg/mL). The LB agar assay plates used for testing A. tumefaciens susceptibility were incubated at 28°C for 24 to 36 h. The assessment of antibacterial activity was based on the measurement of the diameter of the zone of inhibition formed around the disk minus the size of the disk. Three independent trials were conducted for each concentration of each antibiotic. A two-way analysis of variance (ANOVA) was used to compare the means.

### Nitrocefin assay.

Nitrocefin is a chromogenic substrate for measuring β-lactamase activity. Nitrocefin has an absorbance maximum of 390 nm. Upon hydrolysis of the β-lactam ring by a β-lactamase, the absorbance shifts from 390 nm to 486 nm. By monitoring absorbance (*A*) at 486 nm over time and using Beer’s law (*A* = ε*lc*) (where ε is the molar extinction coefficient, *l* is the path length, and *c* is the concentration), we directly measured the rate of β-lactamase hydrolytic activity. The indicated strains were grown in LB until the desired optical density (OD_600_) of 0.6 was reached. The cells were then pelleted by centrifugation at 25,900 × *g* (Fiberlite F14-6 × 250y rotor) for 5 min, and the supernatant was collected and washed three times with phosphate-buffered saline (PBS). Cell lysates were generated by adding BugBuster cell lysis buffer and sonication. No lysozyme or protease inhibitors were added. Cell lysates were normalized based on total protein content (7.5 μg/mL) and volume before incubation in 100 μM nitrocefin solution in a 200-μL reaction volume at room temperature in 20 mM HEPES–300 mM NaCl, pH 7.5. BugBuster lysis buffer was used as a blank. Δ*ampD* lysates were normalized based on total protein content (7.5 μg/mL) and subsequently diluted to 1:5, as the rate of nitrocefin hydrolysis was significantly higher than that of the controls (wild type cells in the absence or presence of AMP 25). Absorbance was immediately measured at 486 nm in 5-s intervals for 300 s (5 min). The change in absorbance (*A*) was converted to change in concentration (*c*) of hydrolyzed product by using Beer’s law (*A* = ε*lc*), where ε is 20,500 M^−1^ cm^−1^ and *l* is 1 cm.

### Construction of *Agrobacterium* strain for plant transformation experiments.

*mltB3* was deleted from the genome of WT Agrobacterium tumefaciens GV3101 by using the same plasmid and technique used to delete the gene in strain C58. The resulting strain, GV3101 Δ*mltB3*, was next transformed by introduction of the empty binary vector pUBQ10-GW (90) by electroporation. This plasmid confers KAN resistance to the *Agrobacterium* strain and also contains tDNA repeat blocks that allow the transfer of glufosinate ammonium (BASTA) resistance into transformed Arabidopsis thaliana. pUBQ10-GW was also electroporated into wild-type GV3101 as a control.

### Arabidopsis thaliana transformation efficiency.

Plants with a bolt height between 2 and 7 cm were transformed by the floral dip method (83). Five plants were transformed on separate days from independent colonies for each C58 and GV3101 strain. Plants were grown in Pro-Mix BX (Premier Tech Horticulture) at 23°C, in 16-h light/8-h dark, 100 to 150 μE m^−2^ s^−1^, and 50 to 70% humidity until the seeds were fully developed. The seeds were collected from fully mature plants and stored at 4°C at low humidity for 1 week. The seeds were then surface sterilized in 15% bleach containing 0.1% Triton X-100 with gentle rocking for 5 min. Seeds were then washed 3 times using sterile water. Seeds were then imbibed in sterile water at 4°C for 3 days. Seeds were then sown into soil using conditions listed above. Once seeds developed true leaves, BASTA (glufosinate ammonium) was applied by spray at a concentration of 10 mg/L. After 4 days, BASTA was reapplied to ensure that only transformed plants survived.

### Determining bacterial load of transformed Arabidopsis thaliana seeds.

Seeds were collected from fully mature transformed plants and stored at 4°C for 1 week with low humidity. Seeds were then surface sterilized in 15% bleach containing 0.1% Triton X-100 with gentle rocking for 5 min. Seeds were then washed 3 times using sterile water. Ten milligrams of seeds for each experimental condition was ground with a sterile mortar and pestle. Ground seeds were then suspended in 1 mL of sterile water and serially diluted. Two hundred microliters of a 10^−1^ dilution was plated on ATGN minimal medium with or without AMP 25. Plates were then incubated at 28°C before colonies were counted.
