# Supplementary material for: Induction of AmpC-Mediated β-Lactam Resistance Requires a Single Lytic Transglycosylase in Agrobacterium tumefaciens
Source: Appl Environ Microbiol. 2022 May 31;88(12):e00333-22. doi: 10.1128/aem.00333-22 (PMC9238390; doi:10.1128/aem.00333-22)
Supplement: Supplemental file 1 — Fig. S1 to S4. Download aem.00333-22-s0001.pdf, PDF file, 2.6 MB [file aem.00333-22-s0001.pdf]

Induction of AmpC-mediated b-lactam resistance requires a single lytic transglycosylase in *Agrobacterium tumefaciens*

Wanda M. Figueroa-Cuilan[1*], Matthew Howell[1&], Christopher Richards[1], Amelia Randich[1^], Akhilesh K. Yadav[2$], Felipe Cava[2], and Pamela J.B. Brown[1#]

[1]Division of Biological Sciences, University of Missouri-Columbia, Columbia, Missouri, 65202
[2]Laboratory for Molecular Infection Medicine, Department of Molecular Biology, Umeå University, Umeå, Sweden

Running title: AmpC-mediated b-lactam resistance of *A. tumefaciens*

[#]Corresponding author:
brownpb@missouri.edu
(573) 884-0384

Present addresses:
*Department of Biological Chemistry, Johns Hopkins University School of Medicine, Baltimore, Maryland 21205
[&]Department of Biology, Westminster College, Fulton, Missouri 65251
[$]Analytical Chemistry Division, CSIR-Indian Institute of Toxicology Research, Lucknow-226001, India
[^]Department of Biology, The University of Scranton, Scranton, Pennsylvania,18510

Contents

Supplemental Figures and Legends 1 – 4

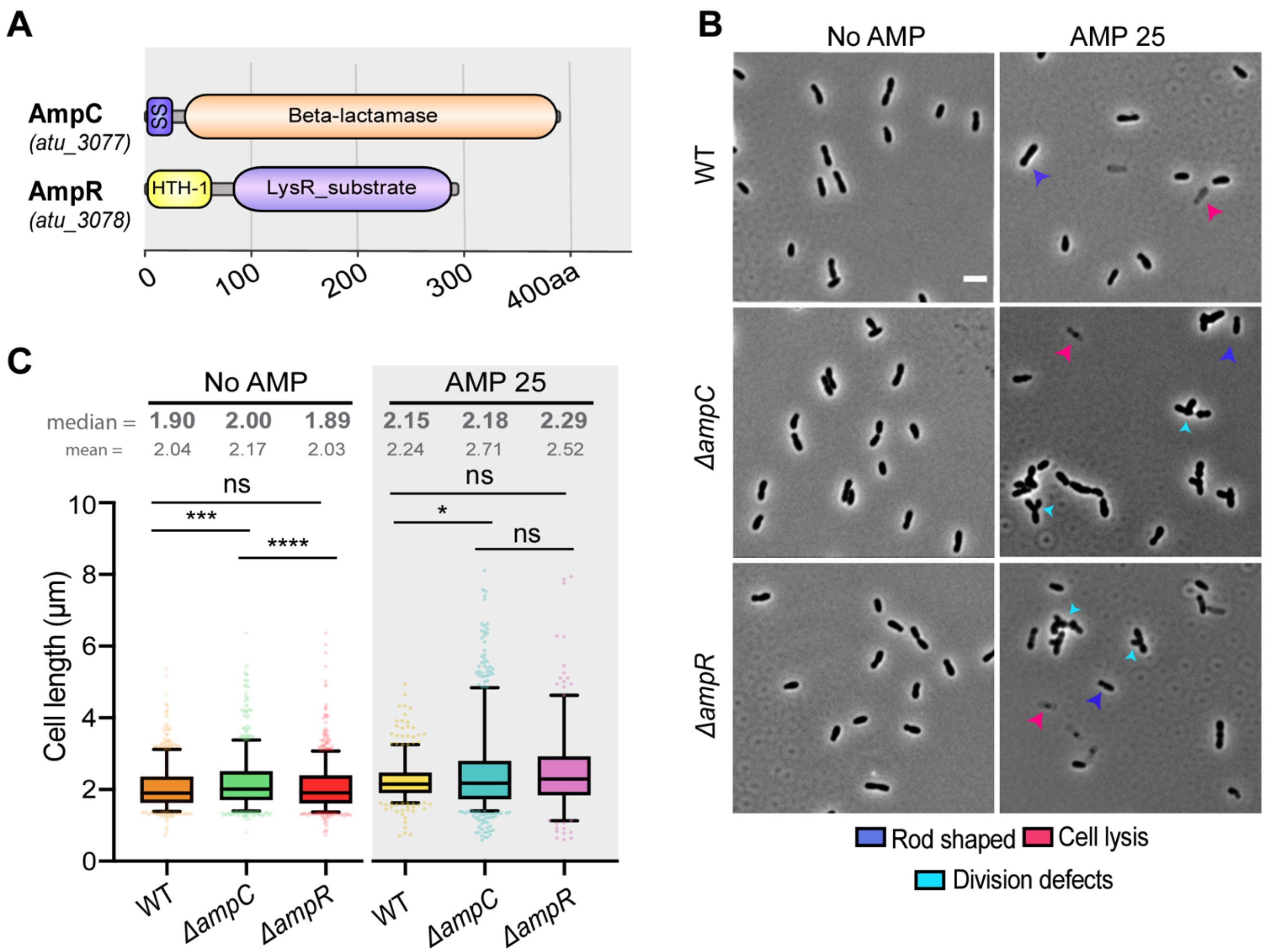

Supplemental 1

SUPPLEMENTAL FIG 1. Putative enzymes involved in ampicillin resistance in the plant pathogen, *A. tumefaciens* C58. (A) Schematic representation of putative proteins involved in ampicillin resistance. Corresponding ORF numbers are indicated under the name of the protein. **(B)** Phase-contrast microscopy of exponentially-growing WT *A. tumefaciens* C58 and *ΔampC* and *ΔampR* incubated in the absence (No AMP) or presence of ampicillin 25 µg/ml (AMP 25) for 2 h. Scale bar = 2 µm. Arrows indicate major phenotypes; Blue = WT/Rod-shaped, magenta = cell lysis, cyan = division defects. **(C)** Box-plots represent the distribution of cell length in WT *A. tumefaciens* C58, *ΔampC,* and *ΔampR* of untreated cells (No AMP) and treated with ampicillin 25 µg/ml (AMP 25) for 2 h before imaging. Top number represents the median cell length. Top quartile (Q1) indicates 75% of the population, middle line represents the median, and bottom quartile (Q2) represents the 25% of the population. Whiskers indicate 5-95% of population. Significance of the cell length distributions are indicated using the non-parametric Kruskal-Wallis test followed by a Dunn posthoc analysis (* = *P <0.1;* *** = *P <0.001;* **** = *P <0.0001;* ns = not significant).

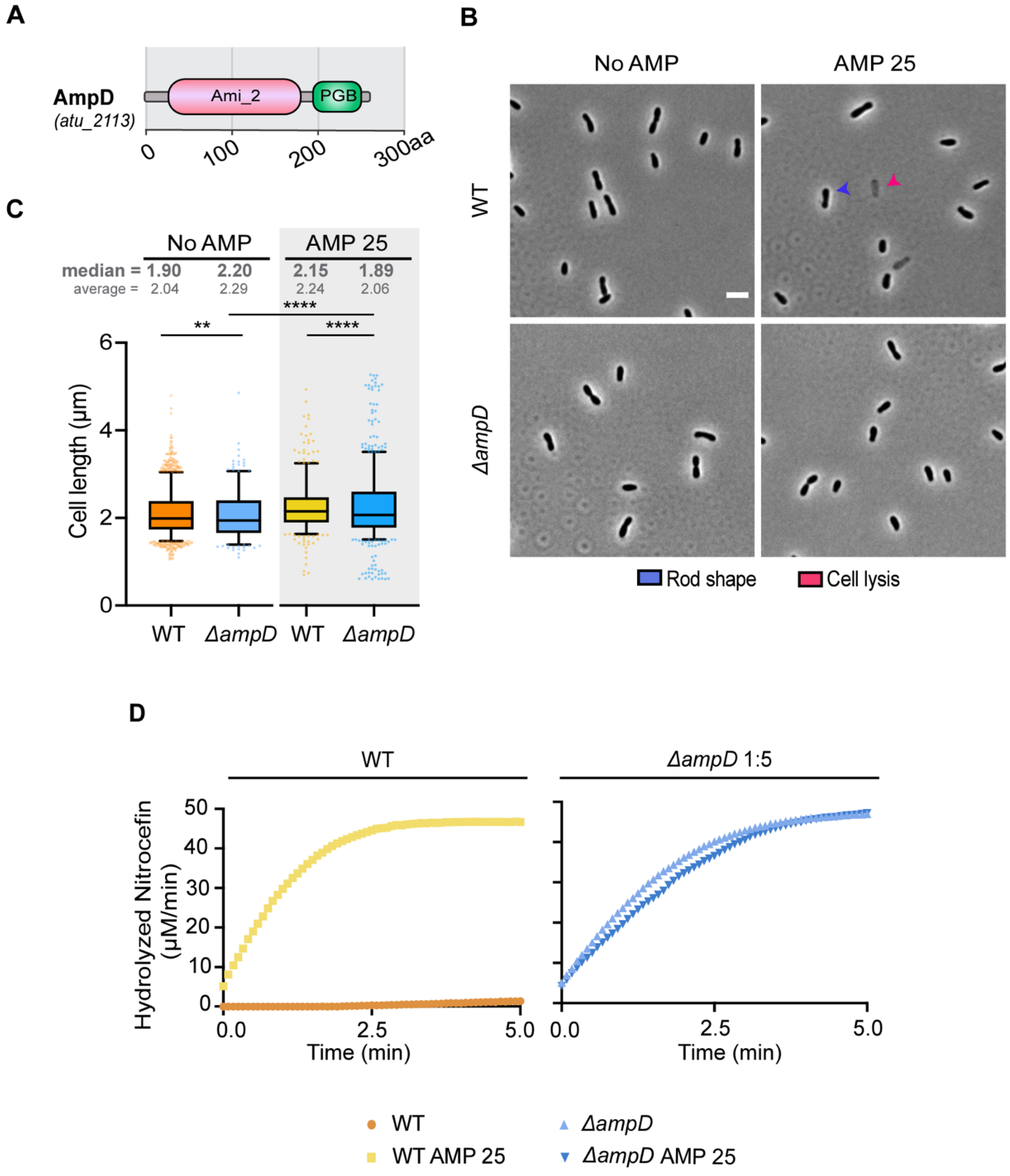

Supplemental 2

SUPPLEMENTAL FIG 2. Loss of AmpD leads to ampicillin resistance. (A) Schematic representation of putative proteins involved in ampicillin resistance. Corresponding ORF numbers are indicated under the name of the protein. **(B)** Phase-contrast microscopy of exponentially growing WT *A. tumefaciens* C58 and *ΔampD* incubated in the absence (No AMP) or presence of ampicillin 25 µg/ml (AMP 25) for 2 h. Scale bar = 2 µm. Arrows indicate major phenotypes; Blue = WT/Rod-shaped, magenta = cell lysis. **(C)** Box-plots represent the distribution of cell length in WT *A. tumefaciens* C58 and *ΔampD* of untreated cells (No AMP) and treated with ampicillin 25 µg/ml (AMP 25) for 2 h before imaging. Distribution of cell lengths was obtained using MicrobeJ (87). Top number represents the median cell length. Top quartile (Q1)

indicates 75% of the population, middle line represents the median, and bottom quartile (Q2) represents the 25% of the population. Whiskers indicate 5-95% of population. Significance of the cell length distributions are indicated using the non-parametric Kruskal-Wallis test followed by a Dunn posthoc analysis (* = $P < 0.1$; ** = $P < 0.01$, *** = $P < 0.001$, **** = $P < 0.0001$). **(D)** Determination of β-lactamase production was performed via a nitrocefin assay using cell lysates. “No AMP” or “AMP 25” indicates cells untreated or treated, respectively, with ampicillin 25 µg/ml for 2 h before the generation of cell lysates. *ΔampD* lysates were normalized based on total protein content (7.5 µg/ml) and subsequently diluted to 1:5 as the rate of nitrocefin hydrolysis was significantly faster than the controls (WT, WT AMP 25) (n=1).

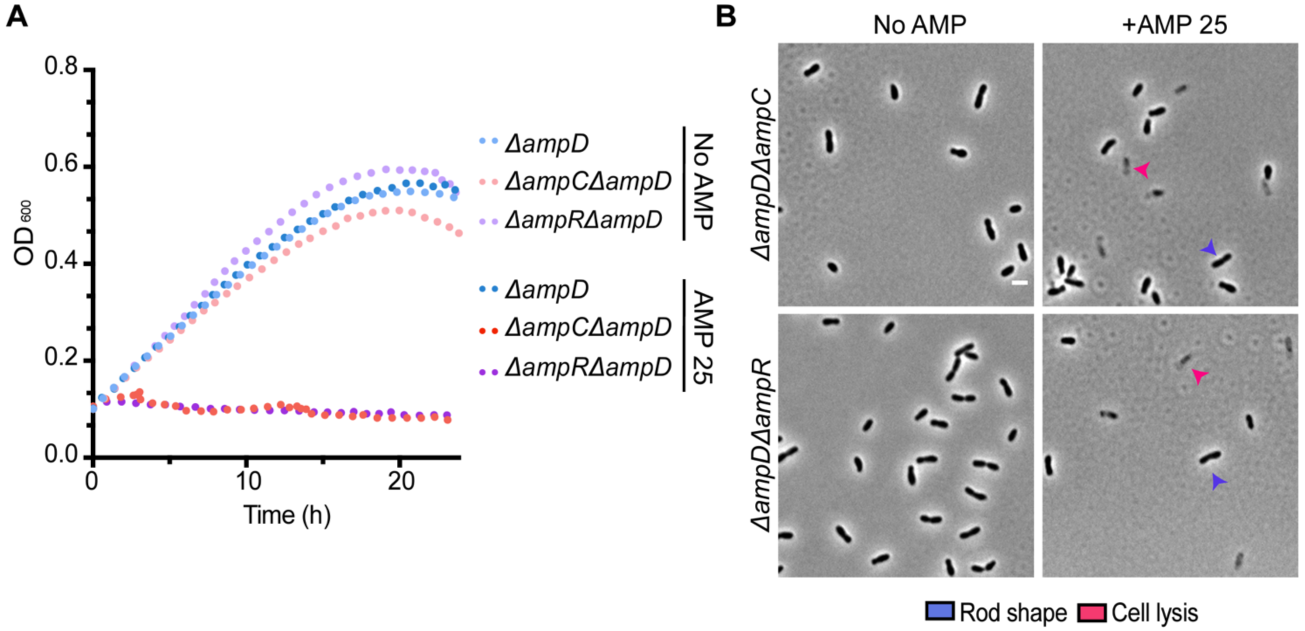

Supplemental 3

SUPPLEMENTAL FIG 3. Loss of AmpD results in constitutive β-lactamase production activity and elevated ampicillin resistance. (A) Growth of *A. tumefaciens* strains in the absence (No AMP) and presence (AMP) of ampicillin 25 μg/ml (AMP 25) for 24 h (n=1, 2 replicates). (**B)** Phase-contrast microscopy of exponentially-growing strains treated with ampicillin 25 μg/ml (AMP 25) for 2 h.

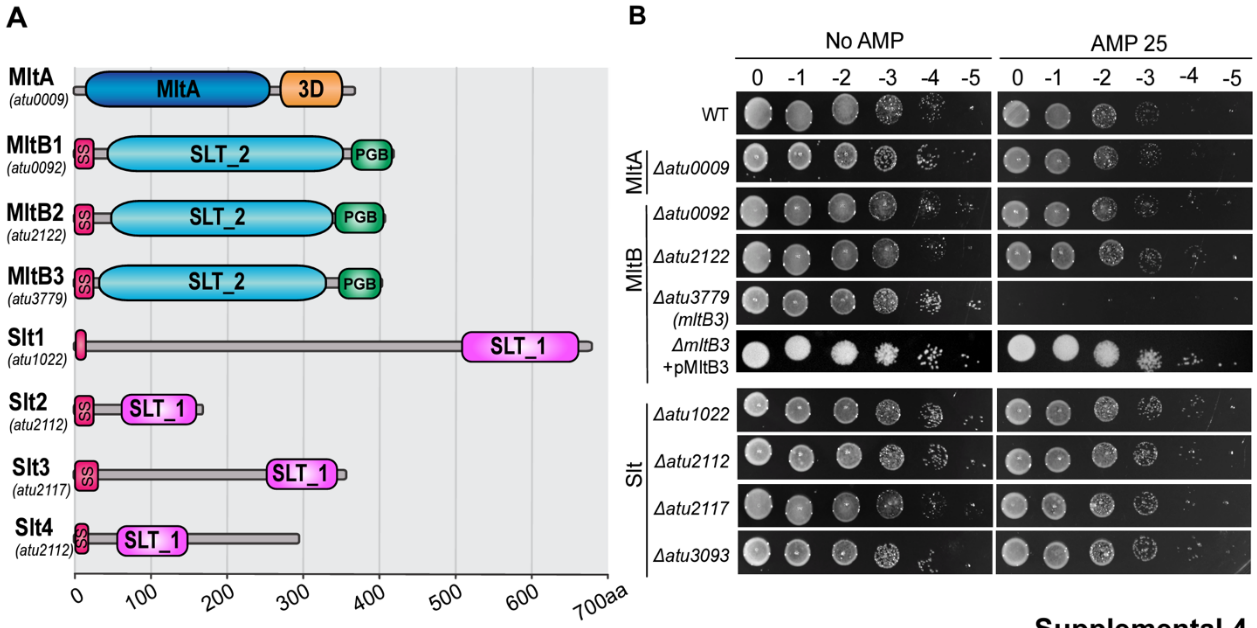

Supplemental 4

SUPPLEMENTAL FIG 4. MltB3 is required for *A. tumefaciens* natural resistance to ampicillin. (A) Domain organization of predicted lytic transglycosylase (LT) proteins. **(B)** Ampicillin susceptibility assay was performed via spotting dilutions. Briefly, indicated strains were cultured ON at 28°C, serially diluted, spotted on solid medium containing no ampicillin (No AMP) or ampicillin 25 µg/ml (AMP 25), and incubated at 28C for 36 h before imaging. Plates used to demonstrate complementation of *ΔmltB3* (*ΔmltB3* +pMltB3) included 1µM IPTG to induce expression of plasmid encoded MltB**.**
